# Premature mortality of gastrointestinal cancer in Iran: trends and projections 2001–2030

**DOI:** 10.1186/s12885-020-07132-5

**Published:** 2020-08-12

**Authors:** Fatemeh Khosravi Shadmani, Farshad Farzadfar, Moein Yoosefi, Kamyar Mansori, Reza Khosravi Shadman, Aliakbar Haghdoost

**Affiliations:** 1 Research Center for Environmental Determinants of Health (RCEDH), Health Institute, Kermanshah University of Medical Sciences, Kermanshah, Iran; 2 Non-Communicable Diseases Research Center, Endocrinology and Metabolism Population Sciences Institute, Tehran University of Medical Sciences, Tehran, Iran; 3 Department of Biostatistics and Epidemiology, School of Medicine , Zanjan University of Medical Sciences, Zanjan, Iran; 4 Student Research Committee, Dezful University of Medical Sciences, Dezful, Iran; 5 HIV/STI Surveillance Research Center, Regional Knowledge Hub, and WHO Collaborating Center for HIV Surveillance, Institute for Futures Studies in Health, Kerman University of Medical Sciences, Kerman, Iran

**Keywords:** Gastrointestinal cancers, Mortality rate, Trend, Projection, Iran

## Abstract

**Background:**

The present study was conducted to determine the trend and projection of premature mortality from gastrointestinal cancers (GI cancers) at national and subnational levels in Iran.

**Methods:**

Employing the data obtained from Iranian Death Registry System (DRS) and population data from census, the mortality rates of GI cancers was calculated among 30–70 age groups. The trends of esophageal, colon and rectum, gallbladder, pancreases, stomach, and liver cancer premature mortalities were estimated and projected at the national and subnational levels from 2001 to 2030. Then, Spatio-temporal model was used to project spatial and temporal correlations.

**Results:**

The overall mortality rate of GI cancers was higher in males than in females, indicating 6.1, 3.9 and 3.9% per 100,000 individuals among males in 2001, 2015 and 2030 respectively and 3.8, 3.1 and 3.7 per 100,000 individuals among females in the same time-frame. The overall mortality rate of GI cancers in males was decreasing until 2015 and will remain stationary into 2030; however, the rate will be increasing among females in both time-frames. Also, there was a considerable variation in the mortality trends of different cancers. Pancreatic, gallbladder, and liver cancers were shown to have an increasing trend while a drop was observed in the mortality rates of stomach, colon and rectum, and esophageal cancers.

**Conclusion:**

Variation of GI cancers patterns and trends around the country indicated that a more comprehensive control plan is needed to include the predicted variations.

## Background

Cancer is one of the main causes of death in the world and in Iran [1, 2]. Gastrointestinal (GI) cancers are considered to be the fourth most common cancers worldwide [3]. GI cancers are the first and second causes of cancer-related deaths among Iranian males and females respectively [4]. GI cancers include a wide range of cancers with different causes. Esophageal, colon and rectum, gallbladder, pancreases, stomach, and liver cancers are the most common GI cancers.

GI cancers may lead to years of life lost from premature death, disability, and increased burden of disease. They have significant socio-economic effects on population. Premature death level is one of the indicators used for studying the impacts of screening programs, early detection, and prognostic factors. Also, it can play an important role in health related decisions made in communities [5]. Planning for future is an inseparable part of cancer control and prevention programs. Although the prediction of the incidence and mortality of cancers is largely associated with uncertainty, these predictions may be employed by health planners in assessment and appropriate allocation of resources, prevention, and treatment. These means of planning, also, enable healthcare policymakers to study the results of interventions made to reduce cancer rates and related consequences. To project future cancer mortalities, especially for GI cancers, many statistical models have been presented over the past few decades. These models often provide reasonable and appropriate predictions when applied to the recent trends [6, 7].

Given the importance of modeling in projecting future cancer mortality especially GI cancers on one hand and the rarity of studies on modeling GI cancers in Iran on another, this study was conducted to evaluate premature mortality trend and projection of GI disorders by 2030 at national and subnational levels in Iran. This evaluation can be used for further health care planning and policy making.

## Methods

### Study area

The trend of premature mortality rate from GI cancers at national and subnational levels was obtained from Death Registry System (DRS) (2001–2015). Premature mortality rate of GI cancers was projected into 2030 using the spatio-temporal model stratified by sex, province and cancer type.

### Death data

Death data was gathered from the DRS. The death registration system records death information based on international classification of diseases 10 (ICD10) and death certificates. More details can be found elsewhere [8, 9]. These data usually include instances of incompleteness and misclassification. This problem was addressed at the Non-Communicable Disease Research Center of Tehran University of Medical sciences (NCDRC); details in this regard are provided elsewhere [8 [9]]. In this study, the recorded death data was derived from the IHME GBD codes (B.1.1, B.1.2, B.1.3, B.1.10, B.1.14, B.1.15), which are equivalent to the C00 to C97 codes in the ICD10. The studied cancers were esophageal, colon and rectum, gallbladder, pancreases, stomach, and, liver.

Premature deaths are referred to deaths occurred at the age of 30–70. Annual cancer data was used in 8 age groups at intervals of 5 years (35–39, 65–69) from 2001 to 2015 stratified by sex and province. Death rate was standardized for age through the direct method.

### Population and covariates data

Demographic data for the model were obtained from statistical censuses and yearbooks. This data were projected into 2030 using spectrum software in moderate scenario. The covariates of wealth index, urbanization, and years of schooling were also extracted from the household and expenditure surveys and were projected (via spline model into 2030) to be used in the modeling process.

### Spatio temporal model

The usual prediction models are not made for including space and time correlations in data. Spatio temporal model was used to project spatial and temporal correlations into 2030. Spatio-temporal model assumes that temporal and spatial residuals provide valuable information which is not directly visible but is systematically observed in time and space. The Moran test was used to evaluate spatial correlation (supplementary 1) [10]. Eq. (1 and (2 show the spatial and temporal weight matrix in Spatio-temporal model, respectively.

1 $$ wi,j=\varsigma \times \frac{wai,j\times wti,l}{\sum \left( wai,j\times wti,j\right)} $$

2 $$ \mathrm{wti},\mathrm{j}={\left(1-{\left(\frac{\mid \mathrm{year}\ \mathrm{i}\hbox{-} \mathrm{year}\ \mathrm{j}\mid }{\arg \max \left(|\mathrm{year}\ \mathrm{i}\hbox{-} \mathrm{year}\ \mathrm{j}|\right)+1}\right)}^{\gamma}\right)}^3 $$

To project, the random effect model was first implemented. In the random effect model, the dependent and independent variables were considered to be mortality rate logit and time respectively. The correlation between the provinces was considered to be autoregressive 1. Then, the covariates of urbanization percentage, wealth index, and number of years of education were entered into the model. In the next step, time and space residuals were added to the projections. we selected a model based on training error (divides the data into two parts – a training set and a validation set; the validation set is used for selecting the best model); details in this regard are provided elsewhere [9, 11].

Finally, mortality rate was obtained which was stratified by province and sex for each cancer. Using Markov Chain Monte Carlo (MCMC) algorithm, simulation was carried out to generate a 1000 iterations and calculate the uncertainty interval. Spatio temporal analysis were performed using SpatioTemporal_1.1.9.tar.gzpackage in R software version 3.5.1 (02-07-2018), (available in: https://cran.r-project.org/src/contrib/Archive/SpatioTemporal).

## Results

The results showed that the mortality rate of gastrointestinal cancers was higher in males than in females. The overall mortality rates for cancers in males were 6.1 (5.2–7.0) and 3.9 (3.4–4.5) per 100,000 individuals in 2001 and 2015 respectively. The corresponding values for females were 3.8 (3.3–4.4) and 3.1 (2.4–2.7) per 100,000 individuals. The mortality rate of gastrointestinal cancers until 2015 was decreasing but it will remain stationary into 2030 (3.9 (3.4–4.5)) among males; whereas, at the same time the rate will be increasing among females (3.7 (3.2–4.2)). It worth mentioning that the trend of each cancer differs from the other.

**Fig. 1:**
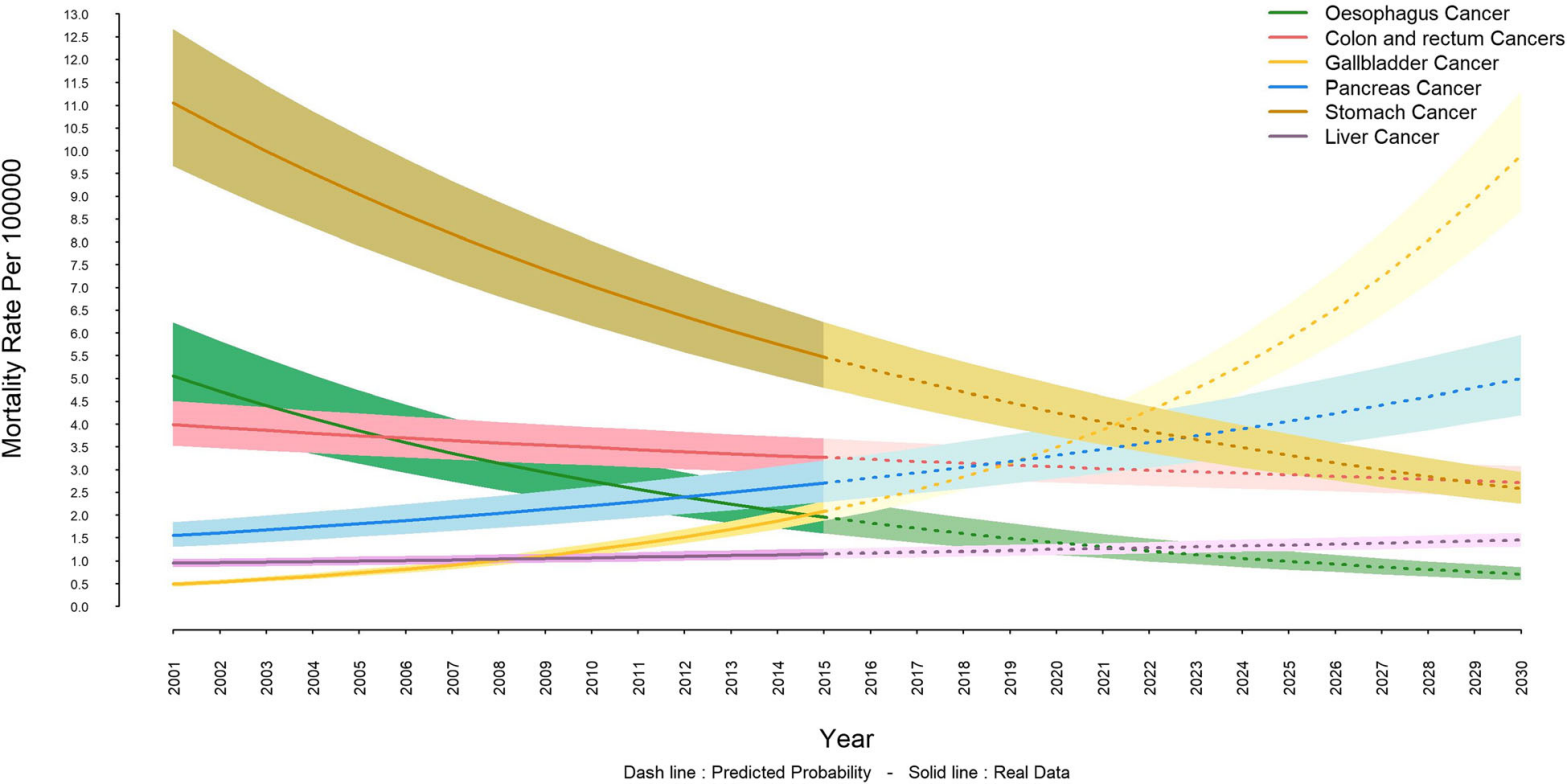
Trend and projection of mortality rate from gastrointestinal cancers in females

**Fig. 2:**
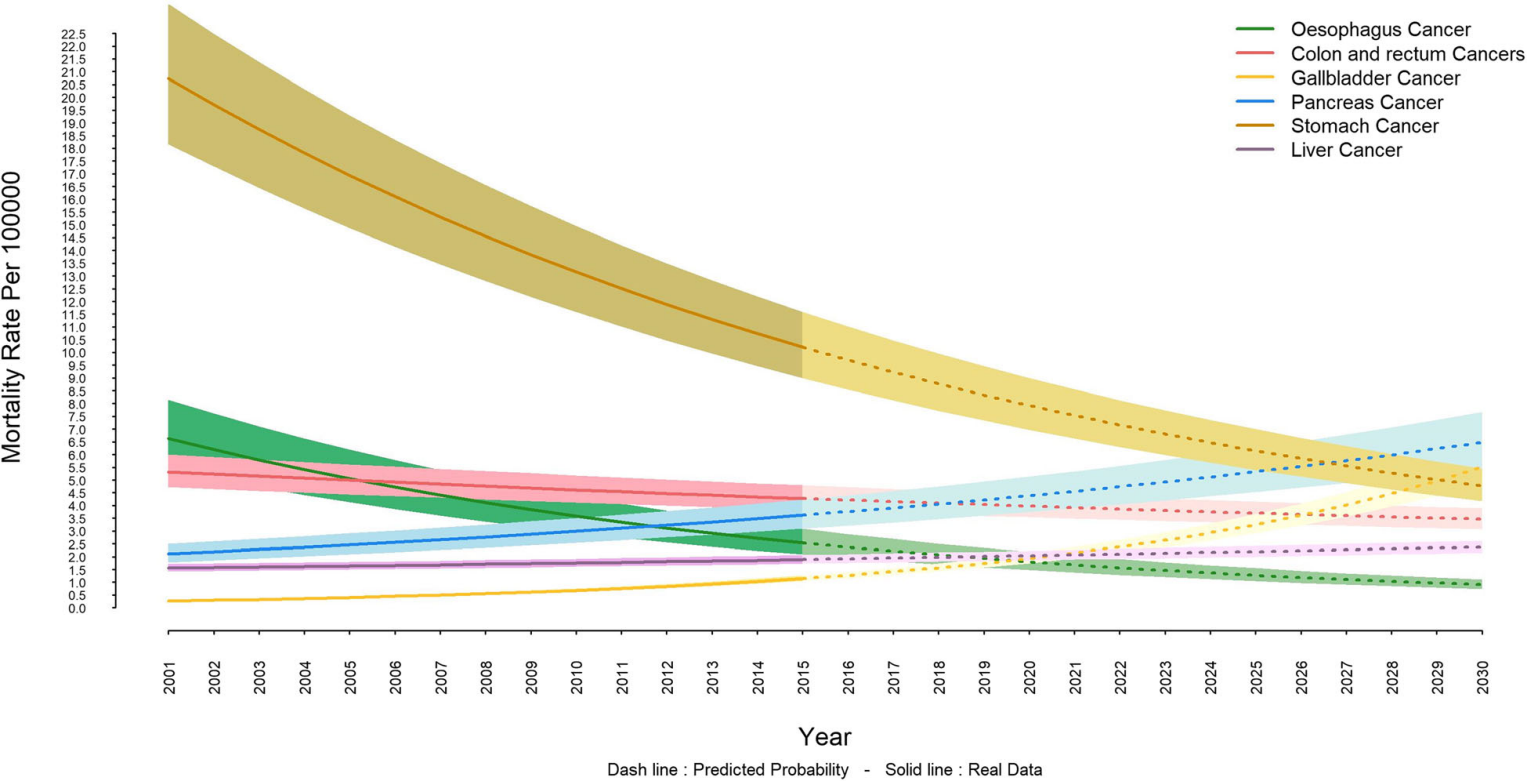
Trend and projection of mortality rate from gastrointestinal cancers in males

The highest mortality rate, in both sexes, is associated with stomach cancer, which its decreasing trend is set to be continued. The mortality rate from stomach cancer in females was 5.4 (6.2–4.7) per 100,000 individuals until 2015, and it is projected to be 2.5 (2.2–2.9) per 100,000 persons by 2030 (Fig. 1). The corresponding values in males are 10.2 (8.9–11.5) and 4.7 (4.1–5.4) per 100,000 people respectively (Fig. 2). Also, esophageal and colon and rectum cancers will have a decreasing trend; whereas, the trends of pancreatic, gallbladder, and liver cancers will be increasing. This pattern is shared by both females and males (Fig. 1 and Fig. 2) and Gallbladder cancer is expected to increase more drastically in females (Fig. 1).

**Fig. 3:**
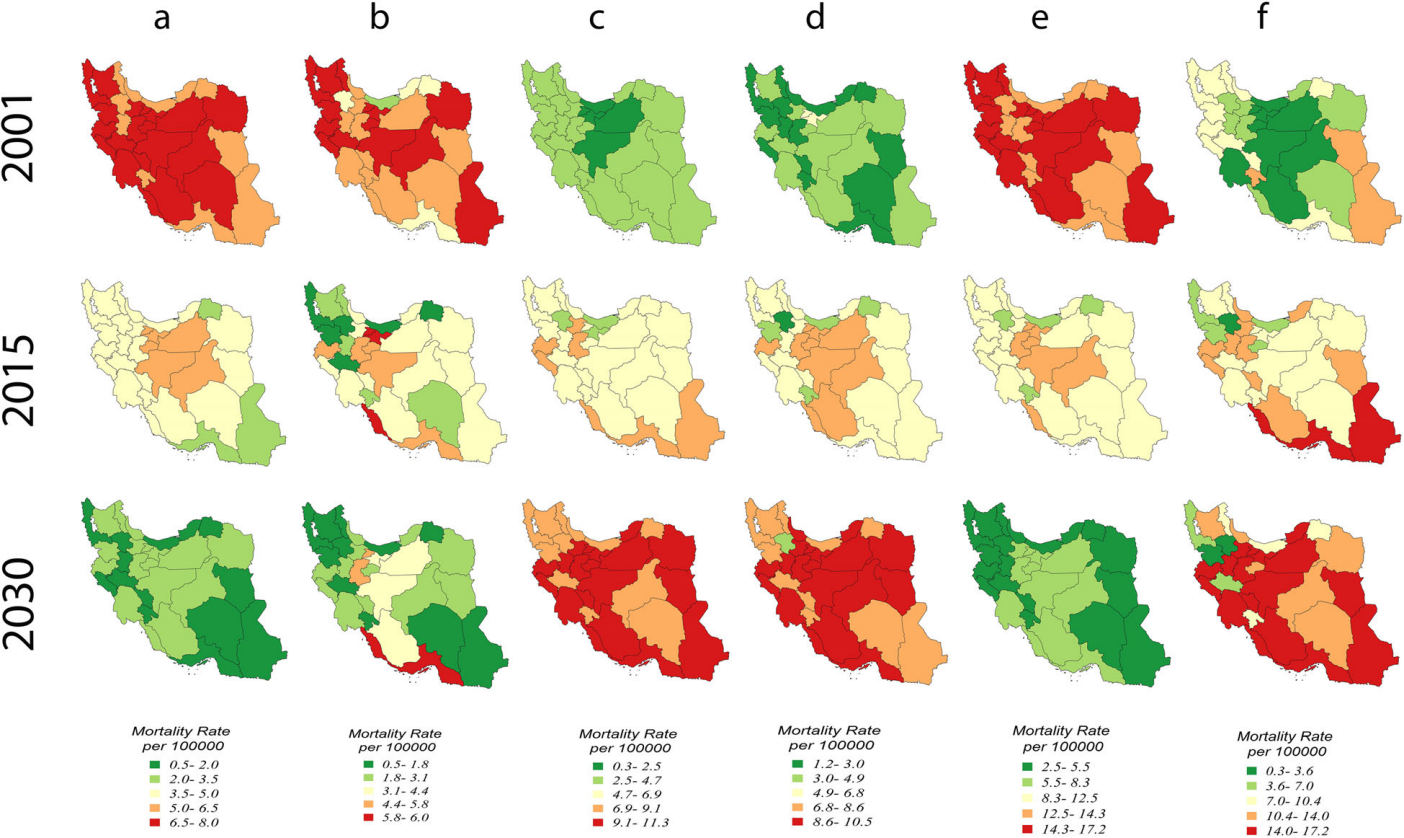
Provincial disparity of gastrointestinal cancers in Iran in 2001, 2015 and 2030 **a** Esophageal cancer, **b** Colon and rectum cancer, **c** Gallbladder Cancer, **d** Pancreases cancer, **e** Stomach cancer, **f** Liver cancer

In 2001, GI cancers were distributed uniformly among the Iranian provinces and hardly indicated any variation; the highest mortality rates of GI cancers were related to esophageal, colon and rectum and stomach and the lowest rates were due to the gallbladder, pancreases and liver cancers. Of course, the distributions were somewhat different and the variation between the provinces was clearer in 2015, indicating that the esophageal, colon and rectum, pancreatic and stomach cancers had the highest mortality rates of GI cancers and were mostly associated with the central provinces of the country. By 2030, GI cancers will, also, be distributed uniformly among the Iranian provinces and no particular variation will be observed. The highest mortality rates of gastrointestinal GI in the projection were observed to be related to gallbladder, pancreases and liver, and the lowest rates were due to esophageal, colon and rectum and stomach cancers (Fig. 3)(the sex-startled map is provided in Fig. 1, Fig. 2, Fig. 3, Fig. 4, Fig. 5, Fig. 6 in supplementary 2).

The mortality rates of esophageal cancer is expected to decrease sharply in all provinces especially southern and southeastern regions in the time-frame of 2001 to 2030. This substantial reduction is also expected for colon and rectum cancer in all provinces especially northwest, south and south east regions. Similarly, a significant reduction is also expected for stomach cancer in all provinces especially west, northwest, east and south east regions in the time-frame of 2001 to 2030. In contrast, the mortality rates of gallbladder cancer is expected to increase sharply in all provinces especially north, west, southwest, east and southeast regions during 2001 to 2030. For pancreases cancer, this substantial increase is also expected in all provinces especially central, west, southwest and east regions. In the same way, this increase is also expected to be significant for liver cancer in all provinces especially north, southwest, east and southeast regions during 2001 to 2030 time-period (Fig. 3).

The mortality rates (stratified by type of the cancers) in each province from 2015 to 2030 are shown in Table 1(sex-stratified rates are provided in Table 1 of supplementary 3).

**Table 1:** The mortality rate and percent of change by type of the cancers in provinces (2015 and 2030)

	Oesophagus Cancer					Colon and rectum Cancer					Gallbladder Cancer					Pancreas Cancer					Stomach Cancer					Liver Cancer				
province	2015 (95% CI)		2030 (95% CI)		% Change	2015 (95% CI)		2030 (95% CI)		% Change	2015 (95% CI)		2030 (95% CI)		% Change	2015 (95% CI)		2030 (95% CI)		% Change	2015 (95% CI)		2030 (95% CI)		% Change	2015 (95% CI)		2030 (95% CI)		% Change
Markazi	2.2	(1.9–2.7)	0.9	(0.7–1.1)	−59.1	4.3	(3.8–4.7)	4.3	(0.8–4.9)	0.0	1.8	(1.6–1.9)	9.1	(0.8–10.4)	405.6	3.3	(2.9–3.8)	7.5	(0.3–8.8)	127.3	8.1	(7.2–9.1)	4.0	(0.5–4.6)	−50.6	1.7	(1.6–1.8)	2.3	(0.1–2.6)	35.3
Gilan	1.9	(1.6–2.3)	0.8	(1.7–1)	−57.9	3.5	(3.1–3.9)	3.0	(1.7–3.4)	−14.3	1.5	(1.4–1.7)	6.8	(1.6–7.7)	353.3	2.8	(2.5–3.3)	5.9	(1.1–6.8)	110.7	7.6	(6.7–8.5)	3.7	(1.3–4.1)	−51.3	1.6	(1.5–1.7)	1.9	(1.7–2)	18.8
Mazandaran	1.7	(1.4–2.1)	0.6	(2.5–0.8)	−64.7	2.3	(2–2.6)	1.7	(2.4–1.9)	−26.1	1.3	(1.1–1.5)	5.4	(2.5–6.5)	315.4	2.0	(1.7–2.5)	3.6	(2.9–4.4)	80.0	6.8	(5.9–7.9)	3.1	(2.7–3.7)	−54.4	1.3	(1.1–1.4)	1.4	(2.2–1.6)	7.7
East Azerbaijan	2.1	(1.8–2.6)	0.8	(3.7–0.9)	− 61.9	3.3	(3–3.7)	2.2	(3.2–2.5)	−33.3	1.6	(1.5–1.8)	7.0	(3.3–7.7)	337.5	2.8	(2.5–3.2)	4.6	(3.4–5.3)	64.3	7.7	(6.9–8.5)	3.5	(3.1–3.9)	−54.5	1.4	(1.4–1.6)	1.6	(3.4–1.7)	14.3
West Azerbaijan	2.0	(1.6–2.4)	0.7	(4.6–0.9)	−65.0	2.7	(2.4–3)	1.5	(4.3–1.7)	−44.4	1.7	(1.5–1.8)	6.9	(4.6–7.9)	305.9	2.3	(2–2.6)	3.3	(4.8–3.9)	43.5	7.3	(6.5–8.1)	3.2	(4.8–3.6)	−56.2	1.3	(1.3–1.5)	1.3	(4.2–1.4)	0.0
Kermanshah	2.1	(1.8–2.6)	0.8	(5.6–0.9)	−61.9	4.1	(3.7–4.5)	3.0	(5.7–3.3)	−26.8	1.9	(1.7–2)	8.7	(5.8–9.7)	357.9	3.2	(2.8–3.6)	5.4	(5.7–6.2)	68.8	7.9	(7.1–8.9)	3.7	(5.3–4.1)	−53.2	1.7	(1.6–1.8)	2.0	(5.8–2.1)	17.6
Khuzestan	2.2	(1.9–2.7)	0.8	(6.7–1)	−63.6	3.7	(3.3–4.1)	3.3	(6.3–3.7)	−10.8	1.7	(1.6–1.9)	8.4	(6.6–9.3)	394.1	3.0	(2.7–3.5)	6.1	(6.3–7)	103.3	7.9	(7–8.8)	3.8	(6.4–4.3)	−51.9	1.5	(1.4–1.6)	2.0	(6.9–2.2)	33.3
Fars	2.1	(1.8–2.5)	0.8	(7.6–0.9)	−61.9	3.9	(3.6–4.3)	3.7	(7.3–4.1)	−5.1	1.7	(1.5–1.8)	8.2	(7.3–9.1)	382.4	3.2	(2.8–3.6)	6.5	(7.6–7.5)	103.1	7.9	(7.1–8.8)	3.8	(7.4–4.3)	−51.9	1.6	(1.5–1.8)	2.3	(7.1–2.5)	43.8
Kerman	1.8	(1.5–2.2)	0.6	(8.5–0.7)	−66.7	2.8	(2.5–3.1)	1.6	(8.4–1.9)	−42.9	1.5	(1.4–1.7)	6.5	(8.6–7.5)	333.3	2.3	(2–2.6)	3.2	(8.6–3.9)	39.1	7.2	(6.4–8.1)	3.1	(8.7–3.6)	−56.9	1.4	(1.3–1.6)	1.6	(8.4–1.8)	14.3
Razavi Khorasan	2.2	(1.8–2.6)	0.8	(9.7–1)	−63.6	3.7	(3.4–4.1)	3.0	(9.7–3.3)	−18.9	1.7	(1.6–1.8)	7.9	(9.2–8.7)	364.7	3.1	(2.7–3.5)	5.6	(9.9–6.4)	80.6	7.8	(7–8.7)	3.7	(9.3–4.1)	−52.6	1.5	(1.4–1.7)	1.9	(9.8–2.1)	26.7
Isfahan	2.6	(2.1–3.2)	0.9	(10.7–1.1)	−65.4	4.2	(3.7–4.7)	3.6	(10.1–4)	−14.3	1.7	(1.5–1.8)	9.0	(10.7–10.4)	429.4	3.6	(3–4.3)	6.5	(10.4–7.9)	80.6	8.2	(7.2–9.4)	3.9	(10.4–4.5)	−52.4	1.5	(1.3–1.6)	2.0	(10.8–2.2)	33.3
Sistan and Baluchistan	1.6	(1.3–1.9)	0.5	(11.4–0.7)	−68.8	3.5	(3.1–4)	2.1	(11.9–2.5)	−40.0	2.1	(1.9–2.4)	9.3	(11.1–10.8)	342.9	2.4	(2–2.9)	3.5	(11.9–4.4)	45.8	7.4	(6.5–8.5)	3.3	(11.8–3.8)	−55.4	2.0	(1.8–2.2)	2.1	(11.8–2.3)	5.0
Kurdistan	2.0	(1.7–2.4)	0.8	(12.6–1)	−60.0	2.6	(2.4–2.9)	1.4	(12.3–1.6)	−46.2	1.7	(1.5–1.9)	6.5	(12.5–7.7)	282.4	2.2	(2–2.6)	3.3	(12.8–3.9)	50.0	7.2	(6.5–8.1)	3.2	(12.8–3.6)	−55.6	1.3	(1.2–1.4)	1.1	(12.1–1.3)	−15.4
Hamadan	1.9	(1.6–2.2)	0.7	(13.6–0.8)	−63.2	3.4	(3.1–3.7)	2.8	(13.5–3.1)	−17.6	1.7	(1.6–1.8)	7.7	(13.7–8.6)	352.9	2.6	(2.3–3)	5.1	(13.4–5.9)	96.2	7.5	(6.7–8.4)	3.6	(13.2–4)	−52.0	1.6	(1.5–1.7)	2.0	(13.9–2.2)	25.0
Chaharmahal and Bakhtiari	1.9	(1.5–2.2)	0.7	(14.5–0.8)	−63.2	3.5	(3.2–3.9)	2.8	(14.5–3.1)	−20.0	1.7	(1.6–1.9)	7.9	(14.1–8.8)	364.7	2.7	(2.4–3.1)	4.9	(14.2–5.7)	81.5	7.6	(6.8–8.5)	3.6	(14.1–4)	−52.6	1.7	(1.6–1.8)	2.1	(14.9–2.3)	23.5
Lorestan	1.9	(1.6–2.3)	0.7	(15.6–0.8)	−63.2	2.7	(2.5–3)	1.5	(15.3–1.6)	−44.4	1.6	(1.5–1.7)	6.3	(15.6–7.2)	293.8	2.3	(2–2.6)	3.2	(15.7–3.7)	39.1	7.2	(6.5–8)	3.1	(15.7–3.5)	−56.9	1.4	(1.3–1.5)	1.3	(15.2–1.4)	−7.1
Ilam	2.0	(1.6–2.3)	0.7	(16.6–0.9)	−65.0	3.7	(3.3–4)	3.0	(16.7–3.3)	−18.9	1.8	(1.6–1.9)	7.9	(16.2–8.7)	338.9	2.9	(2.5–3.2)	5.4	(16.7–6.2)	86.2	7.7	(6.9–8.6)	3.6	(16.2–4.1)	−53.2	1.7	(1.6–1.8)	2.0	(16.9–2.2)	17.6
Kohgiluyeh and Boyer_Ahmad	1.7	(1.4–2)	0.7	(17.6–0.8)	− 58.8	2.8	(2.5–3.1)	1.8	(17.6–2.1)	−35.7	1.7	(1.5–1.8)	7.0	(17.2–7.9)	311.8	2.2	(1.9–2.6)	3.7	(17.2–4.4)	68.2	7.1	(6.3–8.1)	3.3	(17.9–3.7)	−53.5	1.5	(1.4–1.7)	1.5	(17.4–1.7)	0.0
Bushehr	2.1	(1.8–2.5)	0.8	(18.7–1)	−61.9	5.4	(4.9–6)	7.1	(18.3–8.1)	31.5	1.9	(1.7–2)	10.1	(18.4–12.1)	431.6	3.9	(3.4–4.5)	10.5	(18.6–12.8)	169.2	8.6	(7.7–9.6)	4.5	(18.9–5.3)	−47.7	2.1	(1.9–2.3)	3.5	(18.1–4)	66.7
Zanjan	1.9	(1.6–2.3)	0.7	(19.6–0.9)	−63.2	1.4	(1.3–1.6)	0.5	(19.4–0.6)	−64.3	1.2	(1–1.4)	3.8	(19.9–5)	216.7	1.5	(1.2–1.8)	1.7	(19.3–2.2)	13.3	6.3	(5.5–7.2)	2.5	(19.1–3)	−60.3	0.9	(0.8–1)	0.6	(19.5–0.7)	−33.3
Semnan	2.3	(1.9–2.8)	0.8	(20.7–1)	−65.2	3.9	(3.5–4.4)	3.6	(20.2–4.1)	−7.7	1.5	(1.3–1.6)	7.1	(20.2–8.1)	373.3	3.4	(2.9–3.9)	6.8	(20.8–8)	100.0	8.0	(7.1–8.9)	3.8	(20.4–4.3)	−52.5	1.5	(1.4–1.6)	2.1	(20.9–2.3)	40.0
Yazd	2.5	(2.1–3)	0.9	(21.7–1.1)	−64.0	3.9	(3.5–4.3)	3.4	(21.3–3.8)	−12.8	1.5	(1.3–1.6)	6.4	(21.6–7.4)	326.7	3.5	(3–4)	6.9	(21.8–8.2)	97.1	8.1	(7.1–9.2)	3.9	(21.4–4.4)	−51.9	1.4	(1.3–1.5)	1.8	(21.6–2)	28.6
Hormozgan	1.6	(1.3–2)	0.6	(22.5–0.8)	−62.5	4.4	(3.9–4.9)	5.6	(22.8–6.4)	27.3	2.0	(1.8–2.3)	11.3	(22.4–13.5)	465.0	2.9	(2.5–3.5)	7.5	(22.1–9.2)	158.6	7.9	(6.9–9)	4.1	(22.5–4.8)	−48.1	2.2	(2–2.4)	3.7	(22.3–4.2)	68.2
Tehran	2.9	(2.3–3.6)	1.0	(23.8–1.2)	−65.5	4.7	(4.1–5.4)	3.8	(23.4–4.4)	−19.1	1.4	(1.2–1.7)	7.8	(23.9–9)	457.1	4.3	(3.5–5.3)	7.3	(23.1–8.7)	69.8	8.5	(7.2–9.9)	4.0	(23.4–4.6)	−52.9	1.4	(1.3–1.6)	2.0	(23.8–2.2)	42.9
Ardabil	2.0	(1.6–2.3)	0.8	(24.6–0.9)	−60.0	3.1	(2.8–3.4)	2.1	(24.9–2.3)	−32.3	1.7	(1.6–1.8)	6.8	(24.1–7.5)	300.0	2.5	(2.2–2.9)	4.3	(24.8–5)	72.0	7.5	(6.7–8.3)	3.4	(24.3–3.8)	−54.7	1.5	(1.4–1.6)	1.5	(24.4–1.6)	0.0
Qom	2.9	(2.3–3.7)	1.0	(25.8–1.2)	−65.5	4.0	(3.5–4.7)	3.2	(25.8–3.7)	−20.0	1.6	(1.4–1.9)	7.6	(25.6–8.7)	375.0	3.6	(2.9–4.5)	6.5	(25.4–7.9)	80.6	8.3	(7–9.8)	3.9	(25.3–4.5)	−53.0	1.3	(1.2–1.5)	1.7	(25.5–1.9)	30.8
Qazvin	2.2	(1.8–2.6)	0.8	(26.7–1)	−63.6	3.9	(3.6–4.4)	3.4	(26.3–3.8)	−12.8	1.8	(1.7–2)	9.5	(26.3–10.9)	427.8	3.1	(2.7–3.6)	5.9	(26.9–7)	90.3	8.0	(7.1–8.9)	3.8	(26.3–4.4)	−52.5	1.6	(1.5–1.8)	2.2	(26.9–2.4)	37.5
Golestan	1.7	(1.4–2)	0.6	(27.5–0.7)	−64.7	3.5	(3.1–3.9)	3.3	(27.9–3.8)	−5.7	1.7	(1.6–1.9)	8.4	(27.3–9.7)	394.1	2.6	(2.2–3.1)	5.5	(27.5–6.6)	111.5	7.4	(6.5–8.5)	3.6	(27.1–4.2)	−51.4	1.8	(1.6–2)	2.5	(27.2–2.8)	38.9
North Khorasan	1.6	(1.3–2)	0.6	(28.5–0.8)	−62.5	2.5	(2.2–2.9)	1.7	(28.5–1.9)	−32.0	1.6	(1.4–1.8)	6.3	(28.4–7.3)	293.8	2.0	(1.7–2.4)	3.4	(28.8–4.1)	70.0	6.9	(6.1–7.9)	3.1	(28.7–3.6)	−55.1	1.5	(1.3–1.6)	1.5	(28.3–1.7)	0.0
South Khorasan	1.7	(1.4–2.1)	0.6	(29.5–0.8)	−64.7	3.6	(3.2–4)	2.8	(29.5–3.2)	−22.2	1.7	(1.6–1.9)	7.3	(29.4–8.3)	329.4	2.7	(2.3–3.1)	5.0	(29.2–6)	85.2	7.5	(6.6–8.5)	3.5	(29.3–4)	−53.3	1.8	(1.6–2)	2.1	(29.8–2.3)	16.7
Alborz	2.8	(2.2–3.5)	1.0	(30.8–1.3)	−64.3	4.8	(4.2–5.6)	4.3	(30.7–4.9)	−10.4	1.5	(1.3–1.7)	7.7	(30.6–9)	413.3	4.2	(3.5–5.2)	8.1	(30.7–10)	92.9	8.5	(7.3–9.9)	4.1	(30.5–4.8)	−51.8	1.5	(1.3–1.7)	2.0	(30.8–2.3)	33.3
Iran	2.2	(1.8–2.7)	0.8	(31.7–1)	−63.6	3.8	(3.4–4.2)	3.1	(31.7–3.5)	−18.4	1.6	(1.5–1.8)	7.7	(31.8–8.8)	381.3	3.2	(2.7–3.7)	5.7	(31.8–6.8)	78.1	7.8	(6.9–8.9)	3.7	(31.2–4.2)	−52.6	1.5	(1.4–1.7)	1.9	(31.7–2.1)	26.7

## Discussion

The present study was designed and implemented to estimate and project premature mortality rate of GI cancers in Iran from 2001 to 2030. The results demonstrated that the mortality rates in all GI cancers are higher in males than in females. Also, it was shown that the trend of mortality rates for GI cancers were decreasing until 2015 but it will remain stationary among males into 2030; whereas by the same year, females will experience an ascending trend in mortality rates. Our study revealed that the highest mortality rate is associated with stomach cancer in both sexes, which its decreasing trend will continue to 2030. Also, esophageal and colon and rectal cancers will have a downward trend; however, the trend of pancreas, gallbladder and liver cancers will be upward for both sexes.

Cancer is still one of the most important public health problems in Iran, claiming tens of thousands of lives each year. Gastrointestinal cancers have been introduced as dangerous and deadly cancers in the country over the past decades [12]. The results of the present study were not consistent with some of the studies carried out in this field. For example, the study by Pourhoseingholi M et al. carried out to assess mortality rates and trends of GI cancers in Iranian population from 1995 to 2003, indicated that the mortality rates of GI cancers were either increasing or slightly stationary from 1995 to 2003; also, their study showed that the mortality rates for all gastrointestinal cancers were higher in males than in females [13]. In the same study, the mortality rate of colorectal cancer demonstrated a modest increase over the period of 1995 to 2003, but stomach and esophageal cancers were undergone a sharp increase in the trends. Plus, the mortality rate of pancreatic cancer decreased slightly during the studied time-period [13].

In contrast, the results of many studies were consistent with those of our study. Some studies performed in European countries have shown that the mortality rates of stomach and colorectal cancers are, in fact, decreasing [14, 15]. In the United States, the incidence and mortality rates of colorectal cancer have been showing declining trends; also, the mortality rates from this cancer in the European Union have been decreasing as well; however, its incidence and mortality have been increasing in Eastern Europe [16, 17]. The study done by Ana Ferro et al. to determine the worldwide trends in stomach cancer mortality from 1980 to 2011 and its prediction until 2015, indicted that the patterns and trends of stomach cancer mortality have been declining in most countries over the past few years [18]. Another study also showed that stomach cancer related mortality has been decreasing since the 1970s, and the rate is projected to decline in the next decades in Portugal [19]. The study performed by Gaëtan-Romain Joliat et al. to estimate incidence and mortality rates of esophageal, stomach, pancreatic, liver and colorectal cancers by 2030 in Switzerland demonstrated the mortality rates of esophageal, pancreatic and liver cancers will be either stationary or on the slight rise. In contrast, the trend of stomach and colorectal cancer will undergo a significant decrease [20]. As observed, the results of most studies conducted in this area are consistent with those of our study. Generally, reduction of the prevalence of *H. pylori* infection, decrease in tobacco consumption, improvement in socio-economic situation, development of food preserving means, and improvement of screening and detecting methods as well as timely treatment may be the major causes of the decreasing trend of mortality rates of stomach cancer and the other gastrointestinal cancers in most countries around the world [21–24].

Another study conducted in Shanghai to investigate the trend of liver cancer from 1973 to 2012 and project it into 2020. The results showed a 50% decrease in the mortality rate of this cancer by 2012. The decrease was projected to continue until 2020 [25]. In the United States, it has been shown that the mortality rate of liver cancer is increasing and this increase is more prevalent among younger age groups [26] which is consistent with the results of the present study. The increase in this cancer can be explained by the demographic, environmental and lifestyle factors. Young age, gender, exposure to aflatoxin B1, alcohol consumption, smoking, and unhealthy diet are demographic and environmental factors affecting mortality of liver cancer [27]. Also, obesity and diabetes have big impacts on fatty liver and ultimately liver cancer [28, 29]. In a study by the same researchers who carried out the present study, it was shown that obesity and diabetes in Iran are on the rise and this trend will continue in the future which can be one of the factors contributing to the increased number of in liver cancer among Iranian population [30]. Another reason is the lack of vaccination among the age group of 30–70 year olds. Vaccination of hepatitis B in Iran has been included in the vaccination program for the newborns since 1993.

Gallbladder cancer is associated with obesity [31]. Given the increased obesity prevalence in both sexes [30], it can be considered as one of the factors in the increasing trend of gallbladder cancer mortality, especially among females. Since the prevalence of obesity in females is higher than in males, there may be another unknown risk factor in the incidence and mortality of this cancer that should be investigated in further studies.

A study found that smoking is an important risk factor in pancreatic cancer [32]. In another study, the association between diabetes and pancreatic cancer has been confirmed [33]. The trend of smoking in Iran is declining and the increase in pancreatic cancer cannot, directly, be attributed to it. On the other hand, the trend of diabetes is on the rise and it can be considered as a factor in the increasing trend of pancreatic cancer.

The present study indicated that there is geographical variation in different Iranian provinces. Given that studies on the geographic distribution of cancer related deaths in Iran are limited, in the present paper, those studies that address the geographical distribution of the cancer incidences are mentioned. Studies have shown that the north and north east regions of Iran are high-risk areas for esophageal cancer; even one of these provinces (Golestan) has a high incidence at the world level [34, 35]. The causes of high incidence of esophageal cancers in these areas may be traced to drinking hot tea, low intake of fruits and vegetables, poor socio-economic status, and opium consumption [36, 37].

The other study conducted by Khosravi Shadmani F et al. to determine geographic distribution of the incidence of colorectal cancer in Iran, showed that the highest incidence rates of colorectal cancer were found in the central, northern, and western provinces of Iran. Also, generally a wide geographical variation was observed between the Iranian provinces [38]. Studies have shown that colorectal cancer incidence is not distributed uniformly across all geographic units [39, 40]. Some studies have shown that colorectal cancer incidence rate in the north of Iran is higher than in the south of the country [41].

In another study carried-out by Mohebbi M et al. to determine geographical spread of gastrointestinal cancers incidence rates in the Caspian Sea region of Iran, the results showed that that non-random spatial patterns for gastric and esophageal cancers are similar in both sexes. Also, high-incidence clusters were discovered for esophageal, stomach, and colorectal and liver cancers in both sexes. The same study showed that the pancreatic cancer prevalence is low and there was not enough evidence to assess the spatial correlations [42]. In another descriptive study by Sadat Asmarian N et al. conducted to the map stomach cancer rate in Iran using area-to-area Poisson Kriging, the results indicated that the north and northwestern regions of Iran suffer from higher incidence of stomach cancer compared to the deserts and southern regions [43]. Also, another study that amid at spatial analysis of gastrointestinal cancer incidence rate in Iran using Poisson Kriging showed that the north and northwestern regions of the country have higher incidence of gastrointestinal cancer compared to the deserts and southern regions [44].

The comparison of GI cancers mortality rates in the present study with those in the other studies gives an insight into the core of the discussion. In the study by Pourhoseingholi MH et al. to determine the mortality rates and trends of GI cancers in Iran from 1995 to 2004, it was showen that the overall GI cancers mortality rate was increased from 16.06 in 1999 to 19.03in 2003 per 100,000 individuals; this trend underwent a slight decrease in 2004. Also, the rate was higher among males and increased as the age increased. In the study, the highest mortality rate was associated to gastric cancer which was increased from 1.68 in 1999 to 8.78 per 100,000 individuals in 2003; however, the rate was slightly decreased between 2002 and 2004. The mortality rate of CRC cancer moderately increased from 0.46 in 1999 to 3.15 in 2003 per 100,000 individuals; however, this was decreased between 2003 and 2004 as well. Likewise, the rate for esophageal cancer moderately increased from 0.73 in 1999 to 4.28 in 2002 per 100,000 persons but drpped between 2003 and 2004. According to the study, from 2003 onwards, all cancers have shown a decreasing trend [13]. Another study by Salimzadeh H et al. conducted to evaluate the annual trends of GI mortality in Iran during 1990–2015 indicated that the ASMR for gastric, esophagus, liver, and colorectal cancers were 20.5, 5.8, 4.4, and 4.0 per 100,000 persons-years, respectively. Overall, a declining trend was observed for the annual mortality of GI cancers [45]. Additionally, the same declining pattern has been observed in many countries around the world for these cancers [46, 47]. As seen, the results of these studies are consistent with those of the present study. Various studies have suggested that the causes of this annual mortality of GI cancers are due to access to screening or prevention services and changes took place in the cancer risk factors [48].

Mortality trend, in fact, results from combination of incidence and survival trends. Incidence trend reflects changes in the prevalence of risk factors and screening strategies; while survival trend depends on screening strategy and changes in treatment efficacy. In the present study, a dropped was observed in the mortality of stomach, colon and rectum, and esophageal cancers, which may result from changes in the risk factors such as improve in lifestyle, reduction of tobacco use, improve in diet by consumption of high amounts of fresh fruits and vegetables and employ new methods of food preservation. Also, the treatment of Helicobacter pylori infection may play an important role in reducing stomach cancer; some studies have shown that giving antibiotics to patients with Helicobacter pylori infection, may reduce the number of pre-cancerous lesions in the stomach and reduce the risk of developing stomach cancer [49, 50] Additionally, advances in cancer treatment and screening strategies have played a key role in increasing survival rate and reducing the mortality rate from cancers. For instance, studies have shown that after using screening system by testing for occult blood in the feces, deaths from colorectal cancer in many European countries have dropped significantly over the past years [51, 52]. In general, screening programs for gastrointestinal cancers, such as colorectal cancer, may lead to the diagnosis of treatable precancerous lesions and, thereby, reduction in mortality trend. Therefore, the implementation of screening programs in high and medium-risk populations for gastrointestinal cancers should be considered as an important priority for health system policymakers [53].

Today, study of the geographical distribution and estimating the mortality rate are of significant importance to the policy makers and community health planners. Geographic distribution of incidence, prevalence and mortality are key factors in identifying and preventing risk factors. Geographic analysis of disease rates can play an important role in allocating resources, facilities and manpower as well as formulating and evaluating etiological assumptions and interventional measures in areas that require special attention [54]. Therefore, given the limited number of studies conducted in Iran on the geographical distribution of cancer related mortality, further studies are suggested to be performed in this regard. This study is the first investigation carried out at national and sub-national levels to predict the mortality of gastrointestinal cancers in Iran by using corrected and validated mortality data. However, due to changes made in provincial divisions, the researchers faced some problems that were solved by obtaining information at the district level. Another limitation was the lack of information on the incidence of the cancers. Finally, our predictions can be sensitive to the choice of model type and set of assumptions. If the assumptions are not met, the predictions may vary. It should also be noted that this study is part of a larger study aimed at the health impact assessment in Iran. The larger study includes the estimation of 6 risk factor trends and related avoidable deaths. The overall report is hoped to be employed for high-level policymaking in the country.

## Conclusion

The results of this study showed that the overall mortality rate of GI cancers was decreasing until 2015 but it will remain stationary into 2030 in males; whereas, the rate will, still, be increasing in females. There was a considerable variation in the mortality trends of different cancers, showing an increasing trends in mortality of the pancreatic, gallbladder, and liver cancers with drop in the mortality trends in stomach, colon and rectum, and esophageal cancers. Therefore, given the variation in the patterns and trends of GI cancers around the country, a more comprehensive control plan is suggested to include the predicted variations. Finally, although the overall mortality rate from GI cancers is on the rise, some key risk factors are modifiable; therefore, with slight improvement in prevention and intervention policies, a more comprehensive control plan can be developed.

## Supplementary information

**Additional file 1.**

**Additional file 2.**

**Additional file 3.**

## Data Availability

The data is restricted because it is maintained by Ministry of Health and Medical Education (MOHME) of Iran. These information are kept on data servers at the Iranian non-communicable diseases research center (NCDRC) and the researchers are only allowed to extract the results. Since the data are considered nationally confidential, there is no way to access or publish data by third parties. Please contact to: Dr. Farshad Farzadfar Email: f-farzadfar@tums.ac.ir

## Abbreviations

GI: Gastrointestinal cancers
DRS: Death Registry System
ICD10: International Classification of Diseases 10
NCDRC: Non-Communicable Disease Research Center of Tehran University of Medical sciences
MCMC: Markov Chain Monte Carlo
