# Supplementary material for: Premature mortality of gastrointestinal cancer in Iran: trends and projections 2001–2030
Source: BMC Cancer. 2020 Aug 12;20:752. doi: 10.1186/s12885-020-07132-5 (PMC7425152; doi:10.1186/s12885-020-07132-5)
Supplement: Supplementary file 1 — Additional file 1. [file 12885_2020_7132_MOESM1_ESM.docx]

**Supplementary 1**

**The Moran index**

Moran’s index (MI) is known as a common statistical index for identifying spatial autocorrelation, and its values range from -1 to 1. It is obtained from the following equation:

$$I=\frac{N}{\sum_{i} \sum_{j} W_{ij}}\times\frac{\sum_{i} \sum_{j} W_{ij}\left(y_{i}-\overline{y}\right)\left(y_{j}-\overline{y}\right)}{\sum_{i} \left(y_{i}-\overline{y}\right)^{2}}$$

Where **N** is the number of spatial units for which variable **y** is measured and $\mathbf{w}_{\mathbf{ij}}$ is a spatial weight matrix defined to determine the degree of locality [10].

The results showed that the MI is statistically significant for all cancer under study (P-value<0.05), this shows that there is spatial autocorrelation at the level of provinces for mortality all cancers under study. The Table 1 shows the MI and P-Value for mortality of all cancers in 2015.

| Table 1. MI and P-Value for mortality of all cancers in 2015 | | |
| --- | --- | --- |
| Mortality Rate | **MI** | **P-Value** |
| Esophagus cancer | **0.24** | **<0.001** |
| Colorectal cancer | **0.17** | **<0.001** |
| Gallbladder cancer | **0.14** | **<0.001** |
| Pancreas cancer | **0.09** | **0.02** |
| Stomach cancer | **0.28** | **<0.001** |
| Liver cancer | **0.06** | **0.01** |
