# Supplementary material for: Premature mortality of gastrointestinal cancer in Iran: trends and projections 2001–2030
Source: BMC Cancer. 2020 Aug 12;20:752. doi: 10.1186/s12885-020-07132-5 (PMC7425152; doi:10.1186/s12885-020-07132-5)
Supplement: Supplementary file 2 — Additional file 2. [file 12885_2020_7132_MOESM2_ESM.docx]

Supplement 2


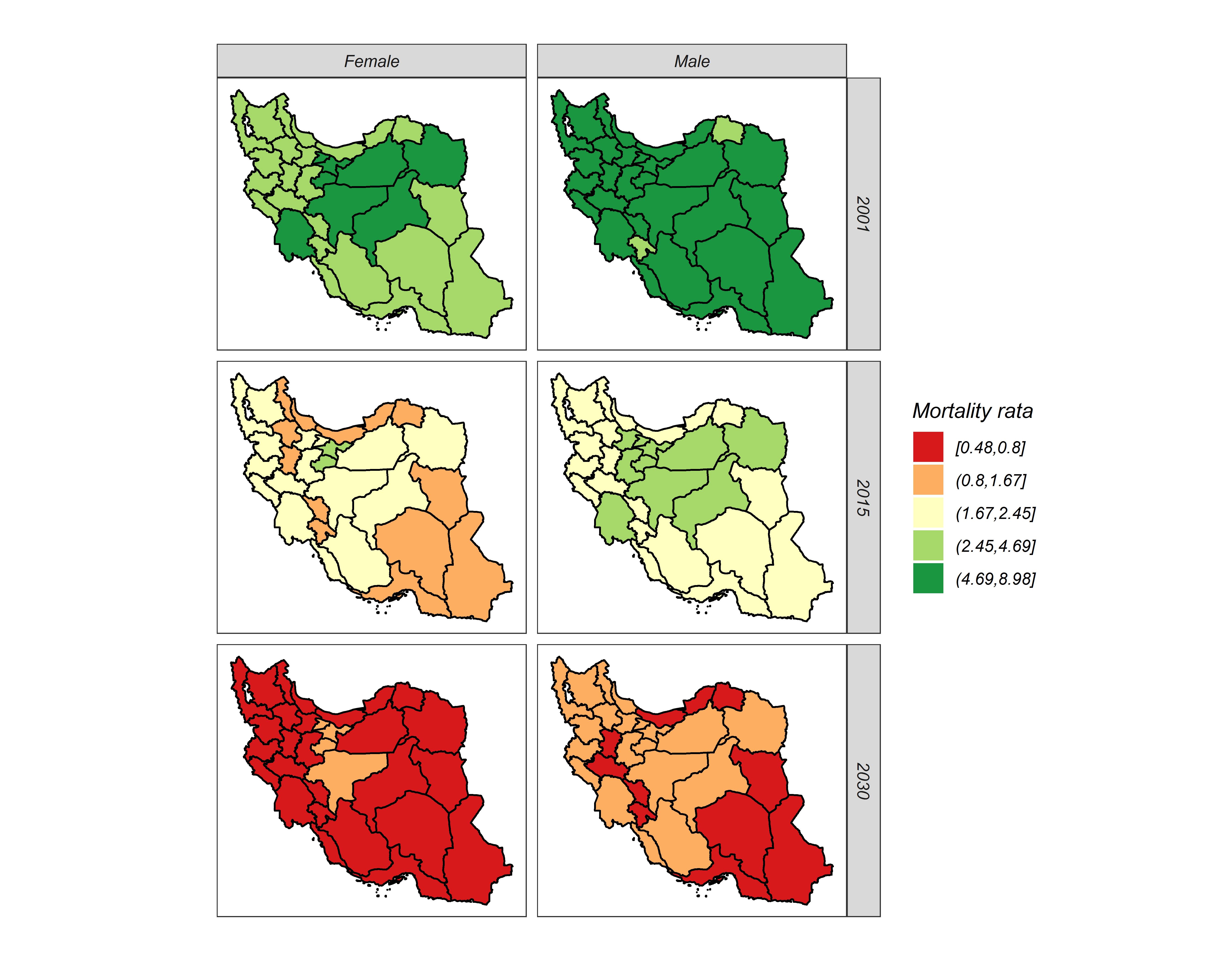


Fig1. Provincial disparity of Esophageal cancer in Iran in 2001, 2015 and 2030, by sex


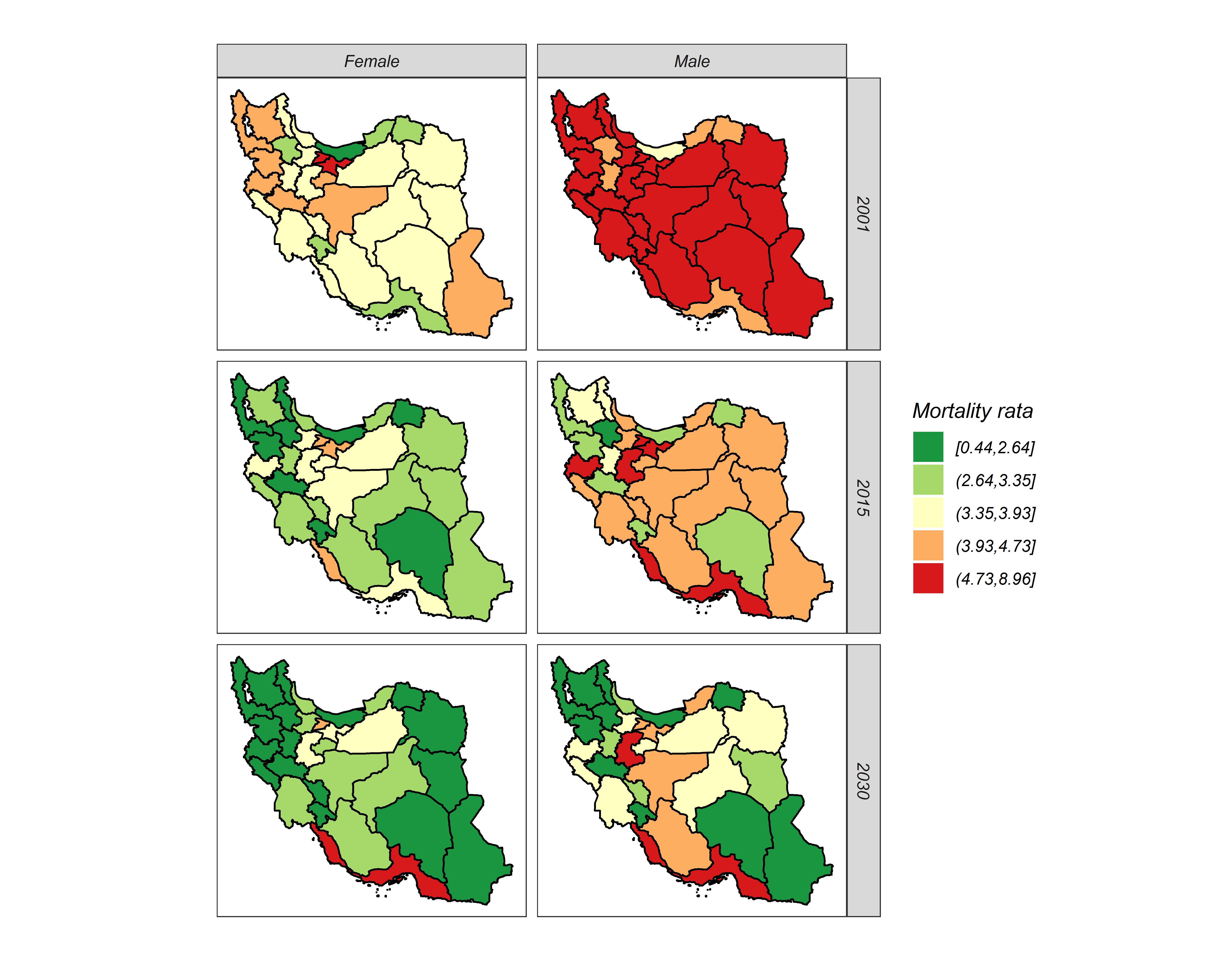


Fig2. Provincial disparity of Colon and rectum cancer in Iran in 2001, 2015 and 2030, by sex


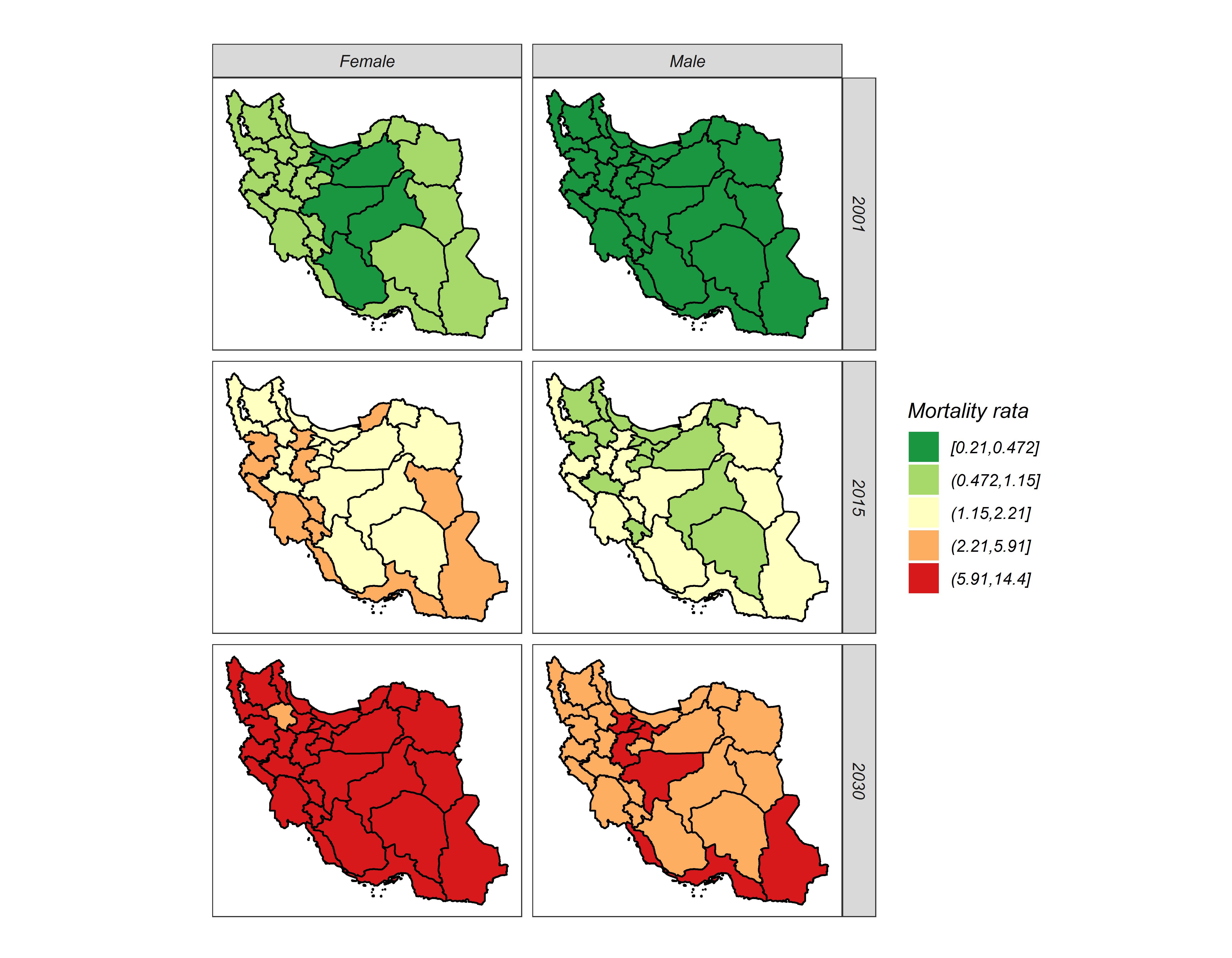


Fig3. Provincial disparity of Gallbladder Cancer in Iran in 2001, 2015 and 2030, by sex


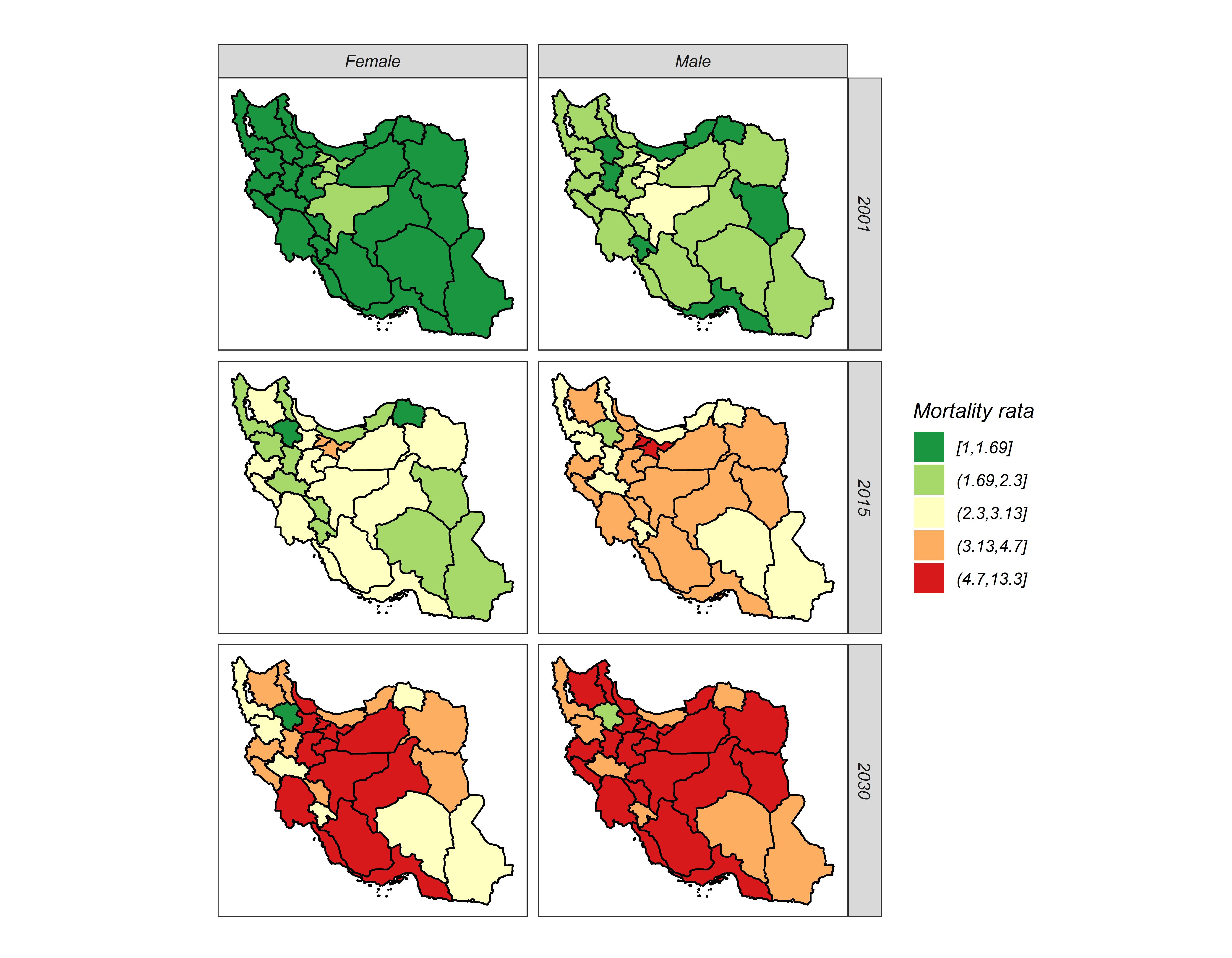


Fig4. Provincial disparity of Pancreases cancer in Iran in 2001, 2015 and 2030, by sex


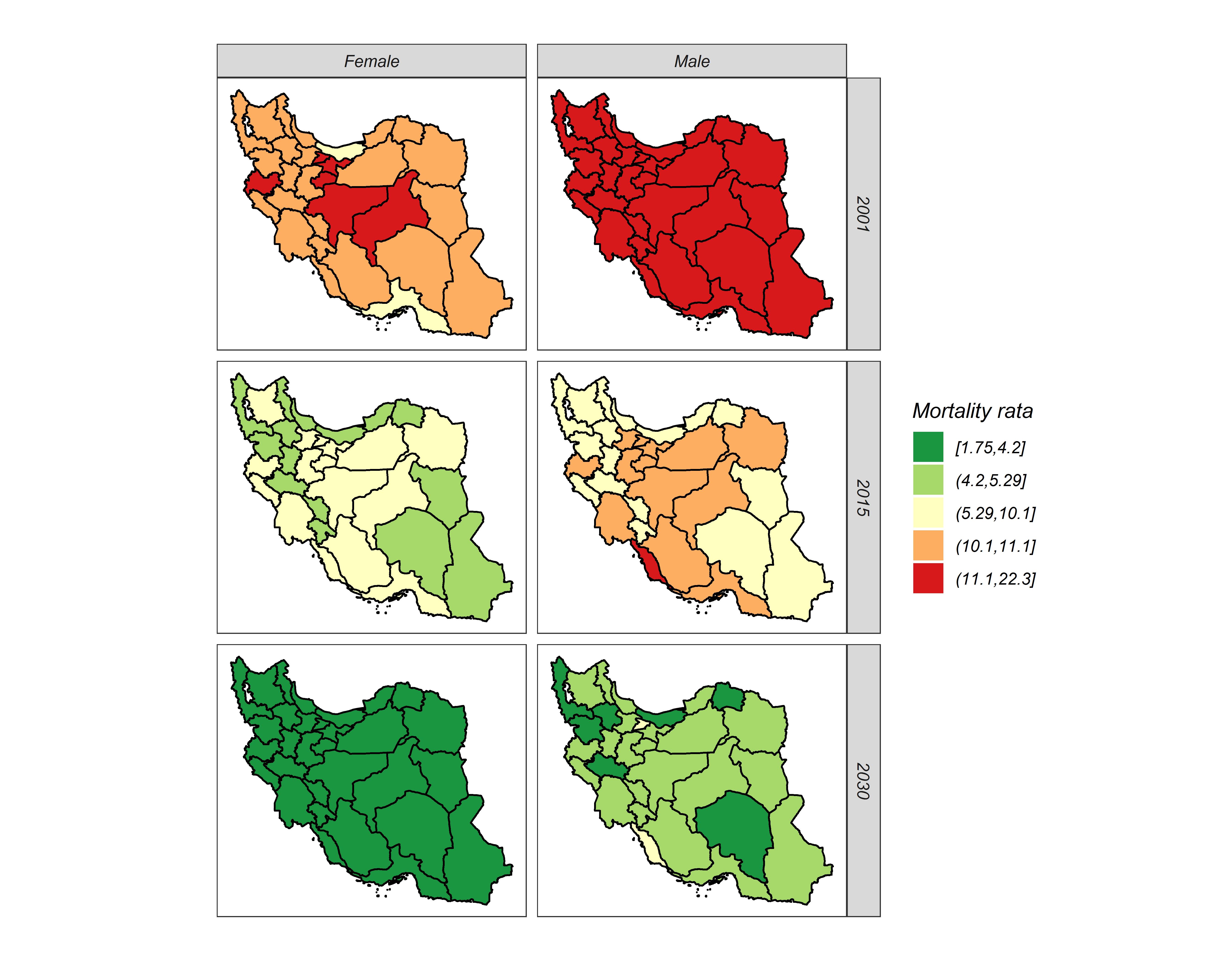


Fig5. Provincial disparity of Stomach cancer in Iran in 2001, 2015 and 2030, by sex


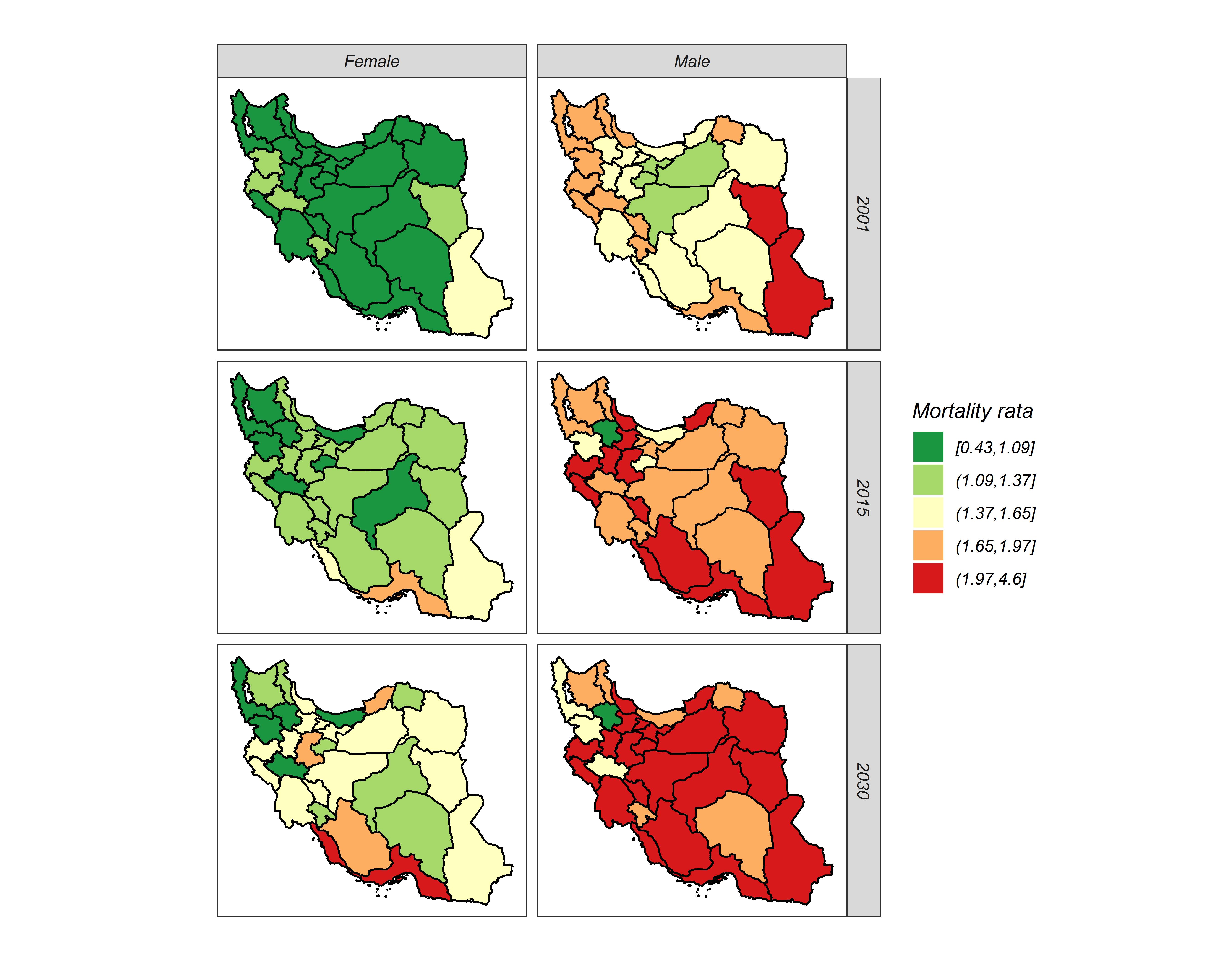


Fig6. Provincial disparity of Liver cancer in Iran in 2001, 2015 and 2030, by sex
