# Supplementary material for: Premature mortality of gastrointestinal cancer in Iran: trends and projections 2001–2030
Source: BMC Cancer. 2020 Aug 12;20:752. doi: 10.1186/s12885-020-07132-5 (PMC7425152; doi:10.1186/s12885-020-07132-5)
Supplement: Supplementary file 3 — Additional file 3. [file 12885_2020_7132_MOESM3_ESM.docx]

Supplement 3

Table 1. The mortality rate by type of the cancers in provinces

|  | Esophageal Cancer | | | | Colon and Rectum Cancer | | | | Gallbladder Cancer | | | | Pancreas cancer | | | | Stomach cancer | | | | Liver cancer | | | |
| --- | --- | --- | --- | --- | --- | --- | --- | --- | --- | --- | --- | --- | --- | --- | --- | --- | --- | --- | --- | --- | --- | --- | --- | --- |
|  | 2015 | | 2030 | | 2015 | | 2030 | | 2015 | | 2030 | | 2015 | | 2030 | | 2015 | | 2030 | | 2015 | | 2030 | |
| province | F | M | F | M | F | M | F | M | F | M | F | M | F | M | F | M | F | M | F | M | F | M | F | M |
| Markazi | 1.9 | 2.5 | .8 | 1.0 | 3.6 | 4.9 | 3.9 | 4.7 | 2.3 | 1.3 | 11.4 | 6.8 | 2.8 | 3.9 | 6.7 | 8.2 | 5.6 | 10.6 | 2.9 | 5.2 | 1.3 | 2.1 | 1.8 | 2.9 |
| Gilan | 1.7 | 2.2 | .7 | .9 | 3.0 | 3.9 | 2.8 | 3.3 | 1.9 | 1.1 | 8.4 | 5.1 | 2.4 | 3.2 | 5.4 | 6.4 | 5.3 | 9.9 | 2.6 | 4.7 | 1.2 | 2.0 | 1.4 | 2.3 |
| Mazandaran | 1.5 | 1.9 | .6 | .7 | 2.0 | 2.6 | 1.5 | 1.9 | 1.7 | .9 | 7.0 | 3.8 | 1.7 | 2.4 | 3.1 | 4.0 | 4.8 | 8.9 | 2.2 | 4.1 | 1.0 | 1.6 | 1.1 | 1.8 |
| East Azerbaijan | 1.8 | 2.4 | .7 | .9 | 2.8 | 3.9 | 1.9 | 2.6 | 2.1 | 1.2 | 9.1 | 4.8 | 2.3 | 3.3 | 3.8 | 5.4 | 5.3 | 10.0 | 2.4 | 4.5 | 1.1 | 1.8 | 1.2 | 2.0 |
| West Azerbaijan | 1.7 | 2.2 | .6 | .8 | 2.3 | 3.1 | 1.3 | 1.8 | 2.2 | 1.2 | 9.1 | 4.6 | 1.9 | 2.7 | 2.7 | 3.9 | 5.1 | 9.5 | 2.2 | 4.1 | 1.0 | 1.7 | 1.0 | 1.6 |
| Kermanshah | 1.8 | 2.4 | .7 | .9 | 3.4 | 4.7 | 2.5 | 3.5 | 2.4 | 1.3 | 11.4 | 5.8 | 2.6 | 3.7 | 4.4 | 6.4 | 5.5 | 10.4 | 2.6 | 4.8 | 1.3 | 2.1 | 1.5 | 2.5 |
| Khuzestan | 1.9 | 2.6 | .7 | .9 | 3.1 | 4.3 | 2.8 | 3.9 | 2.2 | 1.2 | 11.1 | 5.7 | 2.5 | 3.6 | 4.9 | 7.2 | 5.5 | 10.3 | 2.6 | 5.0 | 1.1 | 1.9 | 1.5 | 2.5 |
| Fars | 1.8 | 2.4 | .7 | .9 | 3.3 | 4.6 | 3.1 | 4.3 | 2.1 | 1.2 | 10.7 | 5.6 | 2.6 | 3.7 | 5.3 | 7.7 | 5.5 | 10.3 | 2.7 | 5.0 | 1.2 | 2.0 | 1.7 | 2.9 |
| Kerman | 1.6 | 2.0 | .5 | .6 | 2.4 | 3.1 | 1.5 | 1.8 | 1.9 | 1.1 | 8.2 | 4.8 | 2.0 | 2.6 | 2.9 | 3.5 | 5.0 | 9.3 | 2.2 | 4.0 | 1.1 | 1.8 | 1.2 | 1.9 |
| Razavi Khorasan | 1.9 | 2.5 | .7 | .9 | 3.2 | 4.3 | 2.6 | 3.4 | 2.2 | 1.2 | 10.3 | 5.5 | 2.6 | 3.6 | 4.7 | 6.5 | 5.5 | 10.3 | 2.6 | 4.8 | 1.2 | 1.9 | 1.4 | 2.4 |
| Isfahan | 2.3 | 2.9 | .8 | 1.0 | 3.6 | 4.7 | 3.2 | 3.9 | 2.1 | 1.2 | 11.6 | 6.4 | 3.1 | 4.1 | 5.7 | 7.3 | 5.7 | 10.7 | 2.8 | 5.1 | 1.1 | 1.8 | 1.5 | 2.5 |
| Sistan and Baluchistan | 1.4 | 1.8 | .5 | .6 | 3.0 | 4.1 | 1.8 | 2.4 | 2.7 | 1.5 | 12.2 | 6.3 | 2.0 | 2.8 | 3.0 | 4.1 | 5.2 | 9.8 | 2.3 | 4.3 | 1.5 | 2.5 | 1.6 | 2.6 |
| Kurdistan | 1.7 | 2.3 | .7 | .9 | 2.2 | 3.1 | 1.1 | 1.7 | 2.2 | 1.1 | 8.9 | 4.1 | 1.8 | 2.7 | 2.5 | 4.1 | 5.0 | 9.5 | 2.2 | 4.2 | 1.0 | 1.6 | .8 | 1.4 |
| Hamadan | 1.6 | 2.1 | .6 | .8 | 2.9 | 3.8 | 2.5 | 3.1 | 2.2 | 1.2 | 10.1 | 5.4 | 2.2 | 3.0 | 4.4 | 5.7 | 5.3 | 9.8 | 2.5 | 4.6 | 1.2 | 2.0 | 1.5 | 2.5 |
| Chaharmahal and Bakhtiari | 1.6 | 2.1 | .6 | .7 | 3.0 | 4.1 | 2.4 | 3.2 | 2.2 | 1.2 | 10.0 | 5.8 | 2.3 | 3.2 | 4.2 | 5.6 | 5.3 | 9.9 | 2.5 | 4.6 | 1.3 | 2.1 | 1.6 | 2.6 |
| Lorestan | 1.7 | 2.2 | .6 | .8 | 2.4 | 3.1 | 1.3 | 1.6 | 2.1 | 1.2 | 8.1 | 4.5 | 2.0 | 2.6 | 2.8 | 3.5 | 5.1 | 9.4 | 2.2 | 4.0 | 1.0 | 1.7 | 1.0 | 1.6 |
| Ilam | 1.7 | 2.2 | .6 | .8 | 3.0 | 4.3 | 2.5 | 3.5 | 2.3 | 1.2 | 10.3 | 5.4 | 2.3 | 3.4 | 4.4 | 6.5 | 5.3 | 10.1 | 2.5 | 4.8 | 1.3 | 2.1 | 1.5 | 2.5 |
| Kohgiluyeh and Boyer_Ahmad | 1.4 | 1.9 | .6 | .8 | 2.2 | 3.3 | 1.5 | 2.2 | 2.2 | 1.1 | 9.4 | 4.5 | 1.7 | 2.7 | 2.9 | 4.6 | 4.9 | 9.4 | 2.2 | 4.3 | 1.1 | 1.9 | 1.1 | 1.9 |
| Bushehr | 1.8 | 2.4 | .6 | 1.0 | 4.3 | 6.3 | 5.1 | 9.0 | 2.5 | 1.3 | 13.0 | 7.5 | 3.1 | 4.7 | 7.3 | 13.3 | 5.8 | 11.1 | 2.9 | 6.0 | 1.5 | 2.6 | 2.5 | 4.4 |
| Zanjan | 1.6 | 2.2 | .6 | .8 | 1.2 | 1.6 | .4 | .6 | 1.6 | .9 | 4.9 | 2.7 | 1.2 | 1.7 | 1.4 | 2.0 | 4.4 | 8.2 | 1.8 | 3.3 | .6 | 1.1 | .4 | .7 |
| Semnan | 2.0 | 2.6 | .7 | .9 | 3.5 | 4.4 | 3.5 | 3.8 | 1.9 | 1.1 | 8.7 | 5.4 | 2.9 | 3.8 | 6.4 | 7.2 | 5.6 | 10.3 | 2.7 | 4.9 | 1.1 | 1.8 | 1.6 | 2.5 |
| Yazd | 2.2 | 2.8 | .8 | 1.0 | 3.3 | 4.4 | 2.9 | 3.8 | 1.9 | 1.0 | 8.6 | 4.4 | 2.9 | 4.0 | 5.8 | 7.9 | 5.6 | 10.4 | 2.7 | 5.0 | 1.1 | 1.7 | 1.4 | 2.2 |
| Hormozgan | 1.4 | 1.9 | .5 | .7 | 3.7 | 5.0 | 5.0 | 6.1 | 2.6 | 1.5 | 14.4 | 8.2 | 2.5 | 3.4 | 6.6 | 8.3 | 5.4 | 10.2 | 2.9 | 5.3 | 1.7 | 2.7 | 2.8 | 4.6 |
| Tehran | 2.6 | 3.2 | .9 | 1.0 | 4.3 | 5.2 | 3.6 | 4.0 | 1.8 | 1.1 | 9.7 | 5.9 | 3.9 | 4.7 | 6.9 | 7.7 | 6.0 | 11.0 | 2.8 | 5.1 | 1.1 | 1.8 | 1.5 | 2.4 |
| Ardabil | 1.7 | 2.3 | .6 | .9 | 2.5 | 3.6 | 1.7 | 2.5 | 2.2 | 1.2 | 9.0 | 4.6 | 2.0 | 3.0 | 3.4 | 5.3 | 5.1 | 9.8 | 2.3 | 4.5 | 1.1 | 1.8 | 1.1 | 1.9 |
| Qom | 2.6 | 3.2 | .9 | 1.1 | 3.5 | 4.5 | 2.9 | 3.5 | 2.1 | 1.2 | 9.9 | 5.3 | 3.1 | 4.1 | 5.8 | 7.2 | 5.8 | 10.7 | 2.7 | 5.0 | 1.0 | 1.6 | 1.3 | 2.1 |
| Qazvin | 1.9 | 2.5 | .7 | .9 | 3.4 | 4.5 | 3.1 | 3.7 | 2.3 | 1.3 | 12 | 7.2 | 2.7 | 3.6 | 5.2 | 6.5 | 5.5 | 10.4 | 2.7 | 4.9 | 1.2 | 2.0 | 1.7 | 2.7 |
| Golestan | 1.4 | 1.9 | .5 | .7 | 2.9 | 4.1 | 2.8 | 3.9 | 2.3 | 1.2 | 11.1 | 5.5 | 2.1 | 3.1 | 4.4 | 6.5 | 5.2 | 9.8 | 2.5 | 4.8 | 1.3 | 2.3 | 1.9 | 3.2 |
| North Khorasan | 1.4 | 1.9 | .5 | .7 | 2.1 | 3.0 | 1.3 | 2.0 | 2.0 | 1.1 | 8.3 | 4.2 | 1.7 | 2.4 | 2.7 | 4.2 | 4.8 | 9.2 | 2.2 | 4.2 | 1.1 | 1.8 | 1.1 | 1.9 |
| South Khorasan | 1.5 | 1.9 | .6 | .7 | 3.0 | 4.2 | 2.3 | 3.3 | 2.2 | 1.2 | 9.6 | 4.8 | 2.2 | 3.2 | 4.0 | 6.1 | 5.2 | 9.9 | 2.4 | 4.6 | 1.4 | 2.3 | 1.5 | 2.6 |
| Alborz | 2.5 | 3.1 | .9 | 1.1 | 4.3 | 5.3 | 3.9 | 4.7 | 1.9 | 1.1 | 9.4 | 6.0 | 3.8 | 4.7 | 7.4 | 8.8 | 6.0 | 11.0 | 2.9 | 5.3 | 1.2 | 1.9 | 1.6 | 2.5 |
| Iran | 2.0 | 2.5 | .7 | .9 | 3.3 | 4.3 | 2.7 | 3.5 | 2.1 | 1.1 | 9.9 | 5.5 | 2.7 | 3.6 | 5.0 | 6.5 | 5.5 | 10.2 | 2.6 | 4.8 | 1.2 | 1.9 | 1.5 | 2.4 |
